# Early childcare type and child development at age 3.5 years, data from the French ELFE cohort

**DOI:** 10.1093/eurpub/ckac129.522

**Published:** 2022-10-25

**Authors:** R Gomajee, K Barry, M Melchior

**Affiliations:** Social Epidemiology Research Team, Sorbonne University, Pierre Louis Institute of Epidemiology and Public Health, Paris, France

## Abstract

**Background:**

Early childcare has been linked to child development in some countries. The aim of this study is to evaluate the impact of early childcare in the French context, where children can attend different types of childcare facilities prior to age 3 when they enter kindergarten, and development at age 3.5 years.

**Methods:**

10,683 children from the ELFE French national birth cohort were classified into 4 groups depending on their main type of childcare between birth and age three: childminder (n = 5,014), centre-based childcare (n = 2,583), informal childcare (n = 777) and parents only (n = 2,465). Children's development was measured with the short form of the Child Development Inventory (CDI) via parents-reports at age 3.5 years. The CDI score was transformed into a Development Quotient (DQ) taking into account the child's age, and global developmental delay was defined as DQ < 90. Missing data was imputed by Fully Conditional Multiple Imputation with 10 imputations. Multinomial analyses were carried out adjusted by Inverse Probability Weighting based on Propensity Scores calculated using main selection and confounding variables.

**Results:**

Compared to children who were cared for by parents only, children who were cared for by a childminder or in a centre-based childcare had a higher DQ (103.0 and 104.8 respectively) as well as a lower likelihood of global developmental delay (propensity-score weighted OR = 0.84, [95% CI 0.70-1.01] and propensity-score weighted OR = 0.54, [95% CI 0.44-0.66]) respectively.

**Conclusions:**

In the French context, early centre-based childcare attendance is significantly associated with a lower risk of global child delay. Policies should make centre-based childcare more accessible to a broader number of children.

**Key messages:**

• Early childcare type is linked to child development in France.

• Children in centre-based childhood had a lower likelihood of developmental delay compared to those looked after by parents only.

